# Acute healthcare utilization associated with positive SARS-CoV-2 testing or serology among people experiencing homelessness: A prospective cohort study

**DOI:** 10.1371/journal.pone.0343639

**Published:** 2026-03-04

**Authors:** Lucie Richard, Brooke Carter, Michael Liu, Rosane Nisenbaum, Stephen W. Hwang

**Affiliations:** 1 MAP Centre for Urban Health Solutions, Unity Health Toronto, Toronto, Ontario, Canada; 2 ICES Western, London Health Sciences Research Institute, London, Ontario, Canada; 3 Department of Medicine, Brigham and Women’s Hospital, Boston, Massachusetts, United States of America; 4 Dalla Lana School of Public Health, University of Toronto, Toronto, Ontario, Canada; 5 Strategic Partnerships in Health Excellence, Research, and Engagement (SPHERE), Unity Health Toronto, Toronto, Ontario, Canada; 6 ICES Toronto, Toronto, Ontario, Canada; 7 Department of General Internal Medicine, University of Toronto, Toronto, Ontario, Canada; Centers for Disease Control and Prevention, UNITED STATES OF AMERICA

## Abstract

**Background:**

Current literature drastically underestimates SARS-CoV-2 infection among homeless populations, undermining our understanding of acute healthcare utilization following infection. We quantified acute healthcare utilization associated with robustly measured SARS-CoV-2 infection or reinfection among people experiencing homelessness.

**Methods:**

In this prospective cohort study, we recruited randomly selected individuals experiencing homelessness in Toronto, Canada in summer 2021. Over one year of follow up, participants completed detailed surveys and provided biological samples to test for SARS-CoV-2 infection (RT-PCR and ELISA). Research data were linked to health administrative databases held at ICES, a health administrative data repository. Outcomes were identified using ICD-10 codes and temporal proximity to infection onset, and participants with confirmed infection were compared to participants without infection history.

**Results:**

Two thirds of participants (n = 640) experienced at least one SARS-CoV-2 infection over the observation period; 25% experienced two or more infections. Prior to Omicron dominating infections, coding identified 1.55 (95% CI 1.0–2.5) hospitalizations and 4.39 (3.9–5.9) emergency department visits per 100 person-years; within 30 days of infection onset, rates were 4.07 (95% CI 2.9–5.6) and 13.69 (95% CI 11.4–16.4), respectively. Hospitalization and emergency department rates were 16.8 and 2.4 times higher than during random 30-day periods without infection. After Omicron variants became dominant, coding identified 2.27 (95% CI 1.1–4.5) hospitalizations and 3.97 (95% CI 2.4–6.7) emergency department visits per 100 person-years; within 30 days of infection, rates were 1.29 (95% CI 0.3–3.3) hospitalizations and 5.8 (95% CI 3.6–9.0) emergency department visits per 100-person years. However, post-Omicron rates within 30 days of infection were not significantly higher than for 30-day periods without infection.

**Conclusions:**

Despite extremely high SARS-CoV-2 infection and reinfection rates, people experiencing homelessness in Toronto had relatively low rates of acute healthcare utilization following infection. Future work is needed to ascertain rates of chronic outcomes, like post-COVID condition.

## Introduction

Homelessness is a major public health issue in Canada, affecting at least 80,000 people in Ontario in 2024. [1] People experiencing homelessness often rely on emergency shelters that are crowded, shared, and subject to high turnover, [2] increasing exposure to SARS-CoV-2 infection [3–14] and likelihood of reinfection. [14,15] Combined with elevated rates of chronic illness and unequal access to care, [2] these risks may translate to higher rates of COVID-19-related complications requiring hospital care.

Yet, relatively few studies have examined acute healthcare related to SARS-CoV-2 infection in this population. [11,12,16–24] Some of these were small sample, cross-sectional reports with limited generalizability. [18,20] Many also focused solely on confirmed cases, [16,18,19,23] increasing risk of collider bias. [25] All were conducted in high-resource countries. Most importantly, most existing literature relied on PCR testing to ascertain infection, [11,12,16–19,21–23] despite its known limited coverage among unhoused populations. [14,15,20] In most settings, [26–31] there were restrictions and uneven access to PCR testing, [26] meaning studies relying on this data source also suffered from high risk of sampling bias. The only study, to date, avoiding these limitations is a French study that used combined PCR and study-administered serologic assays to ascertain infection. [24] However, this study was conducted prior to the emergence of Omicron and later variants, which greatly increased SARS-CoV-2 infectiousness while reducing acute complications, at least in the general population [32].

To address these gaps, we examined emergency department (ED) visits and hospitalizations following SARS-CoV-2 infection among a large, representative sample of people experiencing homelessness in Toronto, Canada. Using integrated self-report, PCR and rapid antigen tests, community-based PCR testing and repeated study-administered PCR and serological assay testing, we provide comprehensive and consistent infection and reinfection ascertainment coverage extending into the Omicron period. This approach allowed us to accurately ascertain rates of downstream acute healthcare utilization in a high-resource setting, including oft-missed asymptomatic infections.

## Methods

### Study design and setting

We conducted a prospective cohort study in Toronto, a city in Canada on Treaty 13 territory, using data from the *Ku-gaa-gii pimitizi-win* study [33] linked to administrative data at ICES. [34] *Ku-gaa-gii pimitizi-win*, meaning “life is always/forever moving”, is a spirit name given in ceremony by Elder Dylan Courchene from Anishnawbe Health Toronto. The study followed a representative sample of people experiencing homelessness in 2021–2022. ICES is an independent, non-profit institute whose legal status under Ontario’s health information privacy law allows it to collect and analyze health care and demographic data for health system evaluation and improvement.

Recruitment occurred from June to September 2021, when the Delta variant (B.1.617.2) was replacing Alpha (B.1.1.7) in local infections. [35] By December 2021, Omicron BA.1 had in turn overtaken Delta, rising from <1% to >95% of local cases within a month. [36] Omicron variants predominated for the remainder of the study period. [35] During the study period, Toronto implemented multiple COVID-19 mitigation strategies for people experiencing homelessness, including prioritized vaccine access [37] and extensive use of temporary non-congregate “physical distancing hotel” programs [38,39].

Data in this study were linked using unique encoded identifiers and analyzed at ICES. We followed the Reporting of Studies Conducted Using Observational Routinely Collected Data (RECORD) reporting guidelines (S1 Table A in S1 File). [40] Throughout, this study applies a Western onto-epistemological framework rooted in biomedical and positivist approaches to health.

### Data sources

We used *Ku-gaa-gii pimitizi-win* data to establish our cohort and acquire enhanced sociodemographic information unavailable at ICES. We also used the following databases at ICES (accessed between 01/04/2023 and 02/11/2025): the Registered Persons Database, to link our cohort; the COVID19 Integrated Testing Database, to help ascertain infection exposure; and the Discharge Abstract Database and National Ambulatory Care Reporting System database, to ascertain outcomes. These administrative data sources are used extensively in Canadian health services research and have been found to have excellent performance in identifying acute healthcare encounters.[34] Data sources and quality details are further described in S1 Table B in S1 File.

### Population

MAP’s Survey Research Unit team recruited participants by approaching individuals from randomly selected beds or rooms at 62 shelters, physical distancing hotels, and encampments across Toronto between June 16 and September 9, 2021. To be eligible, individuals had to be experiencing homelessness; be at least 16 years old; and provide informed consent. Participants were re-contacted at 3, 6, 9 and 12 months for follow-up. Observation ended December 31, 2022. Full recruitment procedures and sample size calculation details are in the study protocol [33].

### Exposure

At each of the baseline and follow-up interviews, participants self-reported any history of SARS-CoV-2 infection, including positive PCR or rapid antigen tests. These self-reports were supplemented with administrative records from the COVID-19 Integrated Testing Database, which captured PCR test results obtained in the community. In addition, at each of the baseline and follow-up interviews participants provided a saliva sample, which was tested using standard quantitative reverse transcription polymerase chain reaction (RT-qPCR) [41] to detect active SARS-CoV-2 infection. They also provided a blood sample, either plasma tube or dried blood spot, which was analyzed using enzyme-linked immunosorbent assay [42] to detect SARS-CoV-2 antibodies (spike protein trimer, spike protein receptor-binding protein, and nucleocapsid [NP] antigen) associated with infection or vaccination. For plasma or serum, the combined assays have a sensitivity of 91% and near-perfect specificity [42].

Each method of infection ascertainment has important limitations. Asymptomatic infections are common and often go undetected, [13,14] while self-reported data have recall and social desirability bias issues, especially in highly marginalized groups. [43] Serological testing is affected by waning and variable antibody response. [44] Study-administered PCR testing is limited by fixed follow-up intervals. Finally, community-based PCR testing is by definition unreliable wherever individuals have unequal access to testing, as was true for most of the observation period in Ontario [26–28].

To address these limitations, we created a comprehensive, longitudinal exposure measure by triangulating across all available sources. This method defines infections as any of the following: positive PCR or rapid antigen test (RAT) self-reported by the participant; positive PCR test reported in the COVID-19 Integrated Testing Database; positive study-administered PCR test; or at least two of three anti SARS-CoV-2 antibodies exceeding positivity thresholds from study-collected blood samples. For vaccinated participants without positive PCR/RAT tests, serologic evidence was only classified as positive for infection if there was elevated anti-Nucleocapsid protein, which is the only antibody unaffected by COVID-19 vaccines approved in Canada. [42] This approach was previously shown to identify four times more infections [13,14] and reinfections [15] compared to community-based PCR testing alone.

Reinfections were defined separately to account for the possibility of persistent viral shedding and variable changes in antibody levels after initial infection. Reinfection events were defined as: a positive PCR/RAT test more than 90 days after the last infection; a positive serology result where participants had previously sero-reverted (no longer tested positive); or anti-Nucleocapsid protein levels that increased above the assay’s coefficient of variation after a downward trend in anti-Nucleocapsid levels was previously established. The methodology and justification underlying our identification of reinfections is detailed in prior work [15].

Throughout, the date of infection onset was determined through a hierarchical approach, privileging date information from administrative sources first, study-administered PCR testing second, self-reported sources of any type third, and imputed at random between interview dates for infections solely identified by serological testing [45].

### Covariates

To describe the cohort, we obtained sociodemographic information (including age, gender, race, citizenship status, education level, height and weight to compute body mass index, and key self-reported chronic conditions) from the *Ku-gaa-gii pimitizi-win* baseline survey. We also categorized surveys by level of interviewer confidence in responses. All measures were self-reported and, with the exception of age and comorbidities, unavailable at ICES. Chronic conditions identified in the ICES databases had approximately similar rates, with any differences attributable to lifetime self-report compared to limited lookback at ICES. S1 Table C in S1 File describes each covariate used in the study in detail.

### Outcomes

The primary outcome was a hospitalization related to SARS-CoV-2 infection. The secondary outcome was an emergency department (ED) visit related to SARS-CoV-2 infection. Although ICD-10 code u071 (“COVID-19, virus identified”) showed excellent sensitivity for hospital-based cases of COVID-19 illness in Ontario in 2020, [46] its validity is uncertain in periods without mandated testing, [26–28] amongst asymptomatic infections or infections identified in the community. In particular, during the second half of our observation period, Ontario drastically reduced PCR testing and imposed eligibility criteria for access, [28] such that code u071 is almost certainly insensitive, as well as potentially biased, for at least half of our period of interest.

To address this, we applied two distinct outcome ascertainment approaches. The first, diagnosis code based approach (“diagnostic code method”) defined encounters as those with u071 recorded in any diagnostic field. This method enables comparability with previous studies using similar administrative definitions in our region. [11,12] The second approach (“infection-proximate method”) defined a hospitalization or ED visit related to SARS-CoV-2 infection as any encounter occurring within 30 days of a new SARS-CoV-2 infection or reinfection, as adapted from prior work. [12,16] This method overestimates outcomes by including events not causally related to a SARS-CoV-2 infection, despite their temporal proximity. To address this, we compared outcome rates during infection-proximate periods to rates observed during 30-day periods without evidence of infection, started at a random “non-infection” onset date following the distribution of infection dates. The rate during this non-infection period represents a baseline rate against which to compare the infection period rate.

### Statistical analysis

We summarized participant characteristics overall and by successful linkage to administrative databases. We then provide outcome rates per 100 person-years, with 95% confidence intervals, stratified by ascertainment approach and reporting period (before or after Omicron variants became dominant). 95% confidence intervals were calculated using Byar’s approximation for Poisson-distributed counts. [47] To account for changes in virus characteristics over time, we separately reported outcomes for two phases: a pre-Omicron period, starting from each participant’s baseline until December 31^st^ 2021, and an Omicron-dominant period, beginning December 31^st^ 2021 onward. [36] This distinction reflects evidence that Omicron and later variants, while more infectious, had fewer acute complications in the general population [32].

We suppressed reporting counts <= 5 to protect participant privacy. To determine the association between SARS-CoV-2 infection and hospitalization and ED visits, we estimated rate ratios and 95% CIs using unadjusted modified Poisson regression models with generalized estimating equations (to account for repeated observations within individuals), offset by the log of observation time. Due to low event frequencies, we did not adjust models for covariates. Missing data were minimal (less than 1%); thus, we reported complete-case analysis results only. Throughout, p-values < 0.05 (2-sided) were deemed statistically significant. Analyses were conducted using SAS Enterprise Guide v7.1 (SAS Institute Inc., Cary, NC, USA).

### Ethical review

This study received ethics approval from the Research Ethics Board at Unity Health Toronto (REB# 20–272). Participants were all adults or emancipated minors, and provided written informed consent. All collected data were de-identified by MAP’s Research and Evaluation Services Unit. The authors did not have access to information that could identify individual participants during or after data collection.

## Results

**Fig 1:**
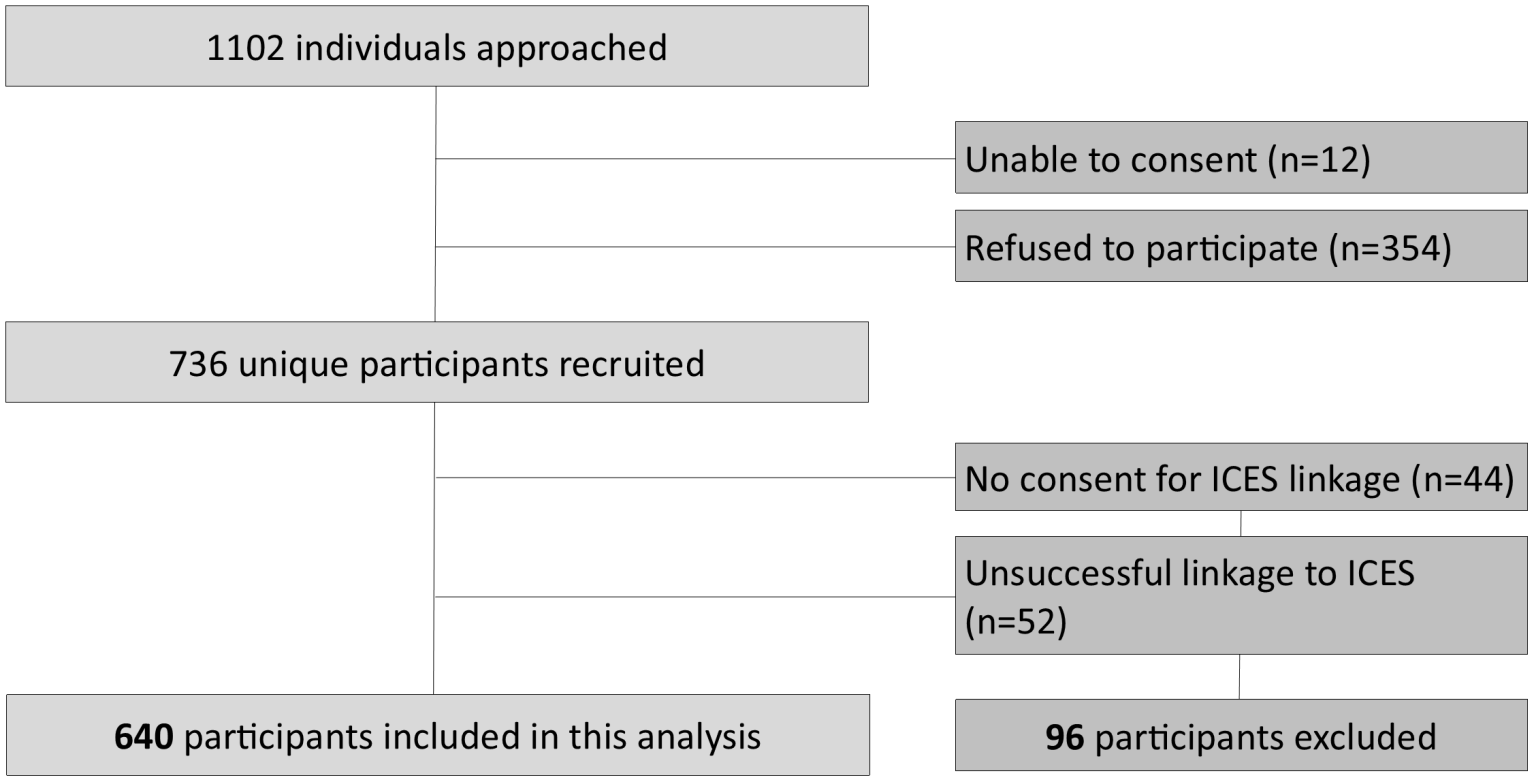
Ku-gaa-gii pimitizi-win cohort inclusion and exclusions.

Of the 736 participants recruited for the study (Fig 1), 44 refused consent for linkage to ICES and 52 were not successfully linked, creating a final sample of 640 participants.

Table 1 compares characteristics of the full cohort against those successfully linked to ICES. Overall, individuals successfully linked were very similar to the full cohort, except for a lower rate of refugees and persons with temporary or other legal status. A majority of participants were 30−49 years old (44.3%) or 50−69 years old (35.7%), self-reported being male (67.4%), White (48.8%), and Canadian citizens (76.6%). Interviewers had very high rates (>95%) of confidence in the reliability of participant survey responses. Over 65% (n = 419) of the cohort experienced at least 1 confirmed SARS-CoV-2 infection during the observation period. More than 25% (n = 163) experienced two or more infection events.

**Table 1:** Ku-gaa-gii pimitizi-win cohort characteristics, overall vs successfully linked to ICES.

	Total (N = 736)	Linked at ICES (N = 640)
Age category, N (%)		
16–29 years	100 (13.59%)	74 (11.56%)
30–49 years	326 (44.29%)	280 (43.75%)
50–69 years	263 (35.73%)	242 (37.81%)
70 + years	47 (6.39%)	44 (6.88%)
Self-reported Gender, N (%)		
Male	486 (66.03%)	438 (68.44%)
Female	231 (31.39%)	185 (28.91%)
LGBTQ2 + /Non-binary/Other/Missing	19 (2.58%)	17 (2.66%)
Self-reported race category, N (%)		
White	359 (48.78%)	332 (51.88%)
Black	159 (21.60%)	122 (19.06%)
Indigenous	28 (3.80%)	28 (4.38%)
Other/multiracial	157 (21.33%)	134 (20.94%)
Refused/Don’t know	33 (4.48%)	24 (3.75%)
Citizenship status, N (%)		
Citizen	564 (76.63%)	520 (81.25%)
Landed immigrant/Permanent Resident	90 (12.23%)	84 (13.13%)
Refugee claimant	55 (7.47%)	24 (3.75%)
Temporary/Other	20 (2.72%)	NR
Refused/Don’t know	7 (0.95%)	<=5
Highest level of education completed, N (%)		
Have not completed High School	208 (28.26%)	188 (29.38%)
High School or Secondary School	255 (34.65%)	223 (34.84%)
Any post-secondary	264 (35.87%)	223 (34.84%)
Refused/Don’t know	9 (1.22%)	6 (0.94%)
Risk factors for severe COVID-19 disease		
Obesity, N (%)	141 (19.16%)	119 (18.59%)
Cancer, N (%)	37 (5.03%)	33 (5.16%)
Chronic Heart Disease, N (%)	46 (6.25%)	42 (6.56%)
Chronic Lung Disease, N (%)	60 (8.15%)	57 (8.91%)
Chronic Kidney Disease, N (%)	18 (2.45%)	16 (2.5%)
3 + Risk factors, N (%)	8 (1.1%)	NR
Interviewer confident in responses received during interview, N (%)	715 (97.15%)	623 (97.34%)
At least 1 confirmed COVID-19 infection over observation period	Not measured	419 (65.47%)
At least 2 confirmed COVID-19 infections over observation period	Not measured	163 (25.47%)

<=5 and NR=Not reportable due to privacy considerations.

Hospitalizations and ED visit rates identified using both infection ascertainment approaches are presented, by period, in Table 2. Using the diagnostic code method (events with ICD-10 code u071), we identified 1.55 hospitalizations (95% CI 1.0–2.5) per 100 person-years and 4.39 ED visits (95% CI 3.9–5.9) per 100 person-years in the pre-Omicron period; while post-Omicron, we identified 2.27 hospitalizations (95% CI 1.1–4.5) per 100 person-years and 3.97 ED visits (95% CI 2.4–6.7) per 100 person-years. Using the infection-proximate method, rates were much higher: pre-Omicron, the rate of hospitalizations and ED visits within 30 days of infection were 4.07 (95% CI 2.9–5.6) and 13.69 (95% CI 11.4–16.4) per 100-person years, respectively; while post-Omicron, we identified a rate per 100 person-years of 1.29 (95% CI 0.3–3.3) hospitalizations and 5.8 (95% CI 3.6–9.0) ED visits.

**Table 2:** Outcomes by type, period and method of ascertainment.

	Pre-Omicron^1^	Post- Omicron^2^
**ICD-10 code “u071” in any diagnostic field**		
Hospitalizations, rate per 100 PYs overall (95% CI)	1.55 (1.0-2.5)	2.27 (1.1-4.5)
ED visits, rate per 100 PYs overall (95% CI)	4.39 (3.9-5.9)	3.97 (2.4-6.7)
**Visits within 30 days of a confirmed COVID-19 infection** ^**3**^		
Hospitalizations, rate per 100 PYs overall (95% CI)	4.07 (2.9-5.6)	1.29 (0.3-3.3)
ED visits, rate per 100 PYs (95% CI)	13.69 (11.4-16.4)	5.81 (3.6-9.0)

CI = Confidence Interval; PY = Person-year ED = Emergency department. 95% confidence interval calculated using the Wilson Score method.

^1^”Pre-Omicron” refers to the period from the individual’s index date to December 31 2021.

^2^“Post-Omicron” refers to the period between January 1 2022 and December 31 2022.

^3^Rates provided per 100 person-years for comparability purposes.

^4^30 days following a date randomly selected from the distribution of dates of confirmed COVID-19 infections for individuals without history of infection.

Table 3 summarizes the results of the modified Poisson models with generalized estimating equations estimating risk of hospitalization or ED visits occurring when someone has a SARS-CoV-2 infection within 30 days, compared to random 30-day periods without infection. Pre-Omicron, SARS-CoV-2 infections within 30 days were associated with an unadjusted rate ratio (uRR) of 16.78 (95% CI 2.3–122.3) for hospitalizations and 2.35 (95% CI 1.6–3.6) for ED visits. Post-Omicron, rates become non-significant: SARS-CoV-2 infections within 30 days were associated with an uRR of 6.1 (95% CI 0.7–54.0) and 1.14 (95% CI 0.6–2.0), respectively.

**Table 3:** Unadjusted modified Poisson regression assessing acute healthcare utilization within 30 days of a COVID-19 infection, or randomly assigned date^1^ for periods without history of infection.

	uRR^a^	95% CI
**Hospitalizations**		
Reference = No evidence of COVID-19 infection in the past 30 days		
COVID-19 infection in the past 30 days (pre-Omicron)	16.78	2.3-122.3
COVID-19 infection in the past 30 days (post-Omicron)	6.10	0.7-54.0
**Emergency Department visits**		
Reference = No evidence of COVID-19 infection in the past 30 days		
COVID-19 infection in the past 30 days (pre-Omicron)	2.35	1.6-3.6
COVID-19 infection in the past 30 days (post-Omicron)	1.14	0.6-2.0

CI = Confidence Interval; ^a^ Unadjusted rate ratio estimated using modified Poisson regression. Models werefitted with generalized estimating equations.

## Discussion

In this representative cohort of people experiencing homelessness in Toronto, Canada, we provide the first estimates of acute hospital-based healthcare utilization following robustly ascertained SARS-CoV-2 infection exposure extending into the Omicron period. Results varied considerably depending on the method used to ascertain events. Using a diagnostic coding approach, we observed 1.6 hospitalizations and 4.4 emergency department (ED) visits per 100-person years pre-Omicron, and 2.3 hospitalizations and 4.0 ED visits per 100 person-years post-Omicron. By contrast, the infection-proximate approach, which captured all events within 30 days of infection, produced higher rates pre-Omicron (4.1 hospitalizations and 13.7 ED events per 100 person-years) but showed marked declines post-Omicron (1.3 and 5.8 events per 100 person-years, respectively). Notably, once compared against randomly selected, non-infection 30-day periods, post-Omicron event rates were *not* significantly higher, suggesting healthcare encounters temporally proximate to infection in this latter period were statistically indistinguishable from routine healthcare behaviours unrelated to SARS-CoV-2 infection.

These differences reflect known limitations of each approach used. The diagnostic coding method assumes comprehensive testing of hospital patients for infection or perfect report of universally-accessible, community-based PCR testing. Throughout our observation period, Ontario lacked these conditions, as policies recommended testing for symptomatic individuals only, [26,27] and then further restricted testing to high-risk groups in January 2022. [28] Consequently, this approach underestimates outcomes. By contrast, the infection-proximate approach overestimates outcomes, as acute care use among people experiencing homelessness is high for a number of reasons unrelated to SARS-CoV-2 infection, and this approach attributes temporally associated but potentially etiologically unrelated events. To address this issue, we compared rates to matched non-infection 30-day periods, which served as a baseline against which to compare rates immediately following infection. The most plausible estimates of acute healthcare utilization related to SARS-CoV-2 infection in this population, in both periods, are therefore the relative risk models in Table 3, which indicates that, within this high-resource policy context, SARS-CoV-2 infection among people experiencing homelessness was associated with an increase in acute healthcare encounters in the pre-Omicron era, but not after Omicron-class variants emerged.

To our knowledge, this is the first study to quantify acute healthcare use following SARS-CoV-2 infection among people experiencing homelessness using adjusted temporal comparisons. Unadjusted outcome rates available elsewhere reflected highly variable sample populations, time periods, testing policies or other pandemic-related response policies relevant to the region. For example, most of the literature estimating hospitalizations [11,12,16–24] ascertained SARS-CoV-2 infections using PCR testing from hospitals, shelters or in the community. Shelter and hospital populations are unrepresentative of the wider population experiencing homelessness, and community-based PCR testing is known to miss a majority of SARS-CoV-2 infections among people experiencing homelessness. [13,14] Wherever incomplete test coverage was also due to testing restrictions (as was true in most studied settings [26–28]), results also may be significantly impacted by selection bias.

Only one prior study, conducted in France (another high-resource setting), similarly used comprehensive, longitudinally-acquired infection ascertainment methods to assess acute healthcare (hospitalization) for COVID-19 among people experiencing homelessness. [24] In that cohort, collected before Omicron variants emerged, [32,36] 3.6% of participants were hospitalized with coding for COVID-19 over a six-month period, with a total all-cause hospitalization rate of 9.9% among infected individuals, representing substantially higher hospitalization frequency than observed in our pre-Omicron results, using either method (1.6 coded and 4.1 temporally proximate events per 100 person-years, respectively).

Several factors may explain differences between our studies. First, the follow-up period of the French study’s time-based approach was much longer (6 months vs 30 days). [24] Second, participants in this other study had much higher levels of morbidity; 14% had three or more risk factors for severe COVID-19 illness, compared to just 1.1% in our cohort. [48] Finally, the French study occurred almost entirely before COVID-19 vaccination was available, and as a result very few people experiencing homelessness would have received vaccines during that study. [49] By contrast, our study began several months after vaccine products became available, and a majority of our cohort (80%) had partial or full vaccine coverage throughout the observation period. [37] Although our event frequency was too low (and our vaccination rates too high) to formally assess the effect of vaccination on outcomes, given known vaccine efficacy in reducing severe outcomes, [50] high vaccine coverage plausibly contributed to our lower observed acute outcome rates.

Overall, our findings underscore the limitations of relying on convenient sources of infection ascertainment information, such as community-based PCR testing, to assess the clinical impact of SARS-CoV-2 infection among marginalized populations. This is consistent with findings in the general population, where serology-estimated infection fatality rates are ten times lower than rates from PCR-estimated infections. [51] In both populations, but particularly among homeless populations, such approaches miss the majority of infections and tend to systematically identify sicker or otherwise unrepresentative individuals, which results in inaccurate estimation of disease virulence. By utilizing a comprehensive infection identification approach that captures the full spectrum of SARS-CoV-2 infections, including often missed asymptomatic or mildly symptomatic cases, our study provides a comprehensive denominator by which to create a revised assessment of the incidence of COVID-19 illness following SARS-CoV-2 infection requiring hospital care.

Future research should prioritize prospective, longitudinally-collected infection ascertainment approaches that provide equal coverage across a representative selection of individuals. In parallel to this, public health interventions for people experiencing homelessness should continue to emphasize prevention of transmission via improved access to stable housing and reduced congregate shelter crowding, as Toronto did with its ‘physical distancing hotel’ program, [38,39] as well as efforts to reduce severe outcomes, particularly through efforts to improve equity of vaccine access and uptake [37].

### Strengths and limitations

This study benefits from several key strengths. First, our sample is broadly representative of people experiencing homelessness in Toronto. Second, we followed participants longitudinally as well as passively through administrative data, employing several methods to comprehensively measure SARS-CoV-2 infection exposure, mitigating issues related to overreliance on any one approach. Third, by analyzing outcomes in the full cohort rather than among infected individuals alone, we avoided collider bias [25] and minimized risk of any residual ascertainment bias.

However, the following limitations should be noted. First, both of the outcome ascertainment methods we used have inherent limitations: diagnostic code-based approaches likely underestimate events, while time-based approaches overestimate them. While our comparison against non-infection periods helped contextualize the latter approach, some residual overestimation may persist. Second, due to low outcome event rates and high vaccine uptake in our cohort, we were unable to directly assess the impact of vaccination or other risk-modifiers on acute healthcare following infection. Third, our study context (a high-resource country that invested significantly in mitigating infection transmission and severe outcomes in this population through vaccination [37] and non-congregate sheltering [39]) means that results may not be generalizable to other regions, particularly those with fewer resources.

## Conclusions

Among people experiencing homelessness in Toronto, acute hospital-based healthcare utilization following SARS-CoV-2 infection was lower than expected, particularly after Omicron variants became dominant, at which point hospitalizations and emergency department visits were no longer significantly higher among infected versus uninfected individuals. This study extends prior work about infectious disease among marginalized populations by highlighting the importance of rigorous infection ascertainment and outcome comparison methods, in particular to adequately capture asymptomatic and mild infections typically missed in conventional approaches. Findings support the importance of public health interventions to mitigate COVID-19 related outcomes among people experiencing homelessness, particularly housing supports and strategies to improve access and uptake of vaccination.

## Supporting information

S1 File Contains the RECORD statement (S1 Table A); Description of administrative sources (S1 Table B); and Variable definitions (S1 Table C). (DOCX)

## Abbreviations

PCR test/ RT-qPCR: Polymerase chain reaction test
RAT: Rapid antigen test
uRR: Unadjusted rate ratio
