# Supplementary material for: Acute healthcare utilization associated with positive SARS-CoV-2 testing or serology among people experiencing homelessness: A prospective cohort study
Source: PLoS One. 2026 Mar 4;21(3):e0343639. doi: 10.1371/journal.pone.0343639 (PMC12959654; doi:10.1371/journal.pone.0343639)
Supplement: S1 File — (DOCX) [file pone.0343639.s001.docx]

**Supporting Information**

**S1 Table A: The RECORD statement^1^ – checklist of items, extended from the STROBE statement, that should be reported in observational studies using routinely collected health data.**

|  | **Item No.** | **STROBE items** | **Location in manuscript where items are reported** | **RECORD items** | **Location in manuscript where items are reported** |
| --- | --- | --- | --- | --- | --- |
| **Title and abstract** | | | | | |
|  | 1 | (a) Indicate the study’s design with a commonly used term in the title or the abstract (b) Provide in the abstract an informative and balanced summary of what was done and what was found |  | RECORD 1.1: The type of data used should be specified in the title or abstract. When possible, the name of the databases used should be included.  RECORD 1.2: If applicable, the geographic region and timeframe within which the study took place should be reported in the title or abstract.  RECORD 1.3: If linkage between databases was conducted for the study, this should be clearly stated in the title or abstract. | Title; Abstract |
| **Introduction** | | | | | |
| Background rationale | 2 | Explain the scientific background and rationale for the investigation being reported |  |  | Introduction |
| Objectives | 3 | State specific objectives, including any prespecified hypotheses |  |  | Introduction, final paragraph |
| **Methods** | | | | | |
| Study Design | 4 | Present key elements of study design early in the paper |  |  | Methods; Study design and setting |
| Setting | 5 | Describe the setting, locations, and relevant dates, including periods of recruitment, exposure, follow-up, and data collection |  |  | Methods; Study design and setting |
| Participants | 6 | *(a) Cohort study* - Give the eligibility criteria, and the sources and methods of selection of participants. Describe methods of follow-up  *Case-control study* - Give the eligibility criteria, and the sources and methods of case ascertainment and control selection. Give the rationale for the choice of cases and controls  *Cross-sectional study* - Give the eligibility criteria, and the sources and methods of selection of participants  *(b) Cohort study* - For matched studies, give matching criteria and number of exposed and unexposed  *Case-control study* - For matched studies, give matching criteria and the number of controls per case |  | RECORD 6.1: The methods of study population selection (such as codes or algorithms used to identify subjects) should be listed in detail. If this is not possible, an explanation should be provided.  RECORD 6.2: Any validation studies of the codes or algorithms used to select the population should be referenced. If validation was conducted for this study and not published elsewhere, detailed methods and results should be provided.  RECORD 6.3: If the study involved linkage of databases, consider use of a flow diagram or other graphical display to demonstrate the data linkage process, including the number of individuals with linked data at each stage. | 6.1 Methods; Data Sources; Recruitment and follow-up  6.2 Methods; Covariates; Supplement 2  6.3 Figure 1 |
| Variables | 7 | Clearly define all outcomes, exposures, predictors, potential confounders, and effect modifiers. Give diagnostic criteria, if applicable. |  | RECORD 7.1: A complete list of codes and algorithms used to classify exposures, outcomes, confounders, and effect modifiers should be provided. If these cannot be reported, an explanation should be provided. | Supplement 1 and 2 |
| Data sources/ measurement | 8 | For each variable of interest, give sources of data and details of methods of assessment (measurement).  Describe comparability of assessment methods if there is more than one group |  |  | Methods: Data sources, Covariates, Outcomes, Statistical Analysis  Supplement 2 |
| Bias | 9 | Describe any efforts to address potential sources of bias |  |  | N/A |
| Study size | 10 | Explain how the study size was arrived at |  |  | N/A (Referred reader to protocol which features sample size calculation) |
| Quantitative variables | 11 | Explain how quantitative variables were handled in the analyses. If applicable, describe which groupings were chosen, and why |  |  | Methods: Covariates and Supplement 2 |
| Statistical methods | 12 | (a) Describe all statistical methods, including those used to control for confounding  (b) Describe any methods used to examine subgroups and interactions  (c) Explain how missing data were addressed  (d) *Cohort study* - If applicable, explain how loss to follow-up was addressed  *Case-control study* - If applicable, explain how matching of cases and controls was addressed  *Cross-sectional study* - If applicable, describe analytical methods taking account of sampling strategy  (e) Describe any sensitivity analyses |  |  | Methods, Statistical Analysis |
| Data access and cleaning methods |  | .. |  | RECORD 12.1: Authors should describe the extent to which the investigators had access to the database population used to create the study population.  RECORD 12.2: Authors should provide information on the data cleaning methods used in the study. | 12.1 Noted work at ICES in Methods: Study Design & Setting  12.2 N/A |
| Linkage |  | .. |  | RECORD 12.3: State whether the study included person-level, institutional-level, or other data linkage across two or more databases. The methods of linkage and methods of linkage quality evaluation should be provided. | Methods: Study Design and Setting |
| **Results** | | | | | |
| Participants | 13 | (a) Report the numbers of individuals at each stage of the study (*e.g.*, numbers potentially eligible, examined for eligibility, confirmed eligible, included in the study, completing follow-up, and analysed)  (b) Give reasons for non-participation at each stage.  (c) Consider use of a flow diagram |  | RECORD 13.1: Describe in detail the selection of the persons included in the study (*i.e.,* study population selection) including filtering based on data quality, data availability and linkage. The selection of included persons can be described in the text and/or by means of the study flow diagram. | 13.1 Figure 1; Results paragraph 1 |
| Descriptive data | 14 | (a) Give characteristics of study participants (*e.g.*, demographic, clinical, social) and information on exposures and potential confounders  (b) Indicate the number of participants with missing data for each variable of interest  (c) *Cohort study* - summarise follow-up time (*e.g.*, average and total amount) |  |  | Results, Paragraph 1 |
| Outcome data | 15 | *Cohort study* - Report numbers of outcome events or summary measures over time  *Case-control study* - Report numbers in each exposure category, or summary measures of exposure  *Cross-sectional study* - Report numbers of outcome events or summary measures |  |  | Results, Paragraph 2 |
| Main results | 16 | (a) Give unadjusted estimates and, if applicable, confounder-adjusted estimates and their precision (e.g., 95% confidence interval). Make clear which confounders were adjusted for and why they were included  (b) Report category boundaries when continuous variables were categorized  (c) If relevant, consider translating estimates of relative risk into absolute risk for a meaningful time period |  |  | Results, Paragraph 2, 3 |
| Other analyses | 17 | Report other analyses done—e.g., analyses of subgroups and interactions, and sensitivity analyses |  |  | N/A |
| **Discussion** | | | | | |
| Key results | 18 | Summarise key results with reference to study objectives |  |  | Discussion paragraph 1,2 |
| Limitations | 19 | Discuss limitations of the study, taking into account sources of potential bias or imprecision. Discuss both direction and magnitude of any potential bias |  | RECORD 19.1: Discuss the implications of using data that were not created or collected to answer the specific research question(s). Include discussion of misclassification bias, unmeasured confounding, missing data, and changing eligibility over time, as they pertain to the study being reported. | Discussion, Limitations section |
| Interpretation | 20 | Give a cautious overall interpretation of results considering objectives, limitations, multiplicity of analyses, results from similar studies, and other relevant evidence |  |  | Discussion, paragraph 1 and 2 |
| Generalisability | 21 | Discuss the generalisability (external validity) of the study results |  |  | Discussion, paragraph 2 and Limitations section |
| **Other Information** | | | | | |
| Funding | 22 | Give the source of funding and the role of the funders for the present study and, if applicable, for the original study on which the present article is based |  |  | Acknowledgements, funding statement |
| Accessibility of protocol, raw data, and programming code |  | .. |  | RECORD 22.1: Authors should provide information on how to access any supplemental information such as the study protocol, raw data, or programming code. |  |

*Checklist is protected under Creative Commons Attribution ([CC BY](http://creativecommons.org/licenses/by/4.0/)) license.

**S1 Table B – Description of administrative data sources**

| Data source | Description |
| --- | --- |
| **ICES Registered Persons Database (RPDB)** | The RPDB database, maintained by the Ontario Ministry of Health, provides demographic information on all individual who have ever received an Ontario health card number, including date of birth and death (if applicable), sex-at-birth, and postal code for each year. This database constitutes the data spine of the linked data framework at ICES and was used to link the *Ku-gaa-gii pimitizi-win* cohort study data. |
| **CIHI Discharge Abstract Database (DAD)** | DAD captures administrative (institution-hospital number, admission category, length of stay, disposition), clinical (diagnoses, procedures, physician) and demographic information (patient gender, date of birth, postal code, county and residence code), on hospital discharges including deaths, sign-outs and transfers. This database contains standardized reporting elements mandated by the Canadian Institute for Health Information and is considered the gold standard source for population-level inpatient hospitalization data in Canada. |
| **National Ambulatory Care Reporting System (NACRS)** | NACRS captures information on patient visits to hospital and community based ambulatory care such as day surgery, outpatient clinics and emergency departments within Ontario. This database contains standardized reporting elements mandated by the Canadian Institute for Health Information and is considered the gold standard source for population-level emergency department data in Canada. |
| **Ontario COVID-19 Integrated Testing Database (C19INTGR)** | C19INTGR, maintained by the Ontario Ministry of Health, is a comprehensive dataset of all available COVID-19 diagnostic laboratory results in Ontario to support efforts to curb the pandemic. Used extensively by the government to inform pandemic-related responses, this database includes data from the Ontario Laboratories Information System (OLIS), distributed testing data from laboratories (DL) within the COVID-19 Diagnostic Network, and Public Health Case & Contact Management (CCM) Solution, formerly known as the integrated Public Health Information System (iPHIS). |
|  |  |

**S1 Table C – Variable definitions**

The table below includes all variables included in this analysis. Covariates are either known from existing literature to affect risk for SARS-CoV-2 infection or are related to SARS-CoV-2 related adverse health outcomes (which may also, indirectly, affect behaviours affected risk of infection). In all instances, measures were collected based on a Settler Canadian (Western) understanding of health-related factors and prevention guidelines appropriate during the COVID-19 pandemic.

| **Variable** | Definition |
| --- | --- |
| Outcome | |
| **Hospitalization related to COVID-19** | Hospital admission in the Discharge Abstract Database with ICD-10 code ‘u071’ in any diagnostic field. |
| **Emergency department visit related to COVID-19** | Emergency department visit in the National Ambulatory Care Reporting System with ICD-10 code ‘u071’ in any diagnostic field. |
| **Hospitalization within 30 days of a COVID-19 infection** | Any hospital admission in the Discharge Abstract Database within 30 days of the estimated onset of a COVID-19 infection |
| **Emergency department visit within 30 days of a COVID-19 infection** | Any emergency department visit in the National Ambulatory Care Reporting System within 30 days of the estimated onset of a COVID-19 infection |
| Exposure | |
| **SARS-CoV-2 infection** | SARS-CoV-2 infection was ascertained at the end of each reporting interval through any of the following sources of evidence:  a) positive PCR or Rapid Antigen Test (RAT) self-reported by the participant;  b) positive PCR test reported in the COVID-19 Integrated Testing Database;  c) positive PCR test administered during an interview; or  d) at least two of three anti SARS-CoV-2 antibodies exceeding positivity thresholds in the blood sample taken during the interview.  For intervals where the participants already had a prior history of SARS-CoV-2 infection, we identified re-infections where  a) positive PCR or RAT tests (from any source) were found more than 90 days after the previous infection began,  b) positive serology results after prior sero-reversion, or  c) positive serology results where anti-Nucleocapsid protein levels increased sufficiently after a downward trend had been previously established, as documented in detail elsewhere.^2^ |
| Other covariates appearing in outputs | |
| **Age category** | Participant’s age as calculated from the ICES Registered Persons Database date of birth and cohort entry date. Expressed as age categories (‘16 to 29 years’; ‘30 to 49 years’; ‘50 to 69 years’; and ‘70+ years’). |
| **Gender** | Participant’s self-reported gender, as of the date of the baseline survey. Answers include ‘male’, ‘female’, ‘LGBTQS2+/Non-binary/Other’ (which also includes ‘gender queer’, ‘agender’, ‘transgender’ or similar) and ‘refused/don’t know’. |
| **Race category** | Participant’s self-reported race identity, as of the date of the baseline survey. Answers include ‘White’, ‘Black’, ‘Indigenous’ (including First Nations, Métis or Inuit), ‘Other/multiracial’ (which includes Arab/Middle Eastern/West Asian, Latin American, East or Southeast Asian, South Asian/Indo Carribean, or where the participant reported multiple racial categories), ‘refused/don’t know’ and ‘missing’. The ‘missing’ category was not included in modelling. |
| **Citizenship status** | Participant’s self-reported citizenship status, as of the date of the baseline survey. Answers include ‘Citizen’, ‘Landed immigrant’ (also known as ‘Permanent resident’), ‘Refugee’, ‘Temporary/Other’ and ‘Refused/Don’t know’. |
| **Immigration history** | Recentness of participant immigration to Canada, as of the date of the baseline survey. Calculated from the Citizenship status and year of immigration variables. Categories include ‘Born in Canada (n/a)’, ‘10 or more years ago’, and ‘Less than 10 years ago’. |
| **Education level** | Participant’s self-reported highest level of completed education, as of the baseline interview date. Answers include ‘less than high school’ (secondary school), ‘high school’, ‘any-post-secondary’ (which combines ‘vocational/technical school’, ‘college/university’, ‘graduate/professional school’), or ‘Refused/Don’t know’. |
| **Obesity** | Participant BMI categorized into the 30+ category, based on participant self-reported height and weight |
| **Cancer** | Presence of participant self-reported history of, or active, cancer |
| **Chronic heart disease** | Presence of participant self-reported chronic heart disease (diagnosed by a physician) |
| **Chronic lung disease** | Presence of participant self-reported chronic lung disease (diagnosed by a physician) |
| **Chronic kidney disease** | Presence of participant self-reported chronic kidney disease (diagnosed by a physician) |
|  |  |
| **Presence of 3+ risk factors for severe COVID-19 disease** | Presence of three or more risk factors for severe COVID-19 disease (any of: obesity, cancer, chronic heart disease, chronic lung disease, chronic kidney disease) |
| **Confidence** | Interviewer level of confidence in the responses provided by the participant |
| **Pre-Omicron period** | Outcomes occurring prior to or on December 31 2021, before Omicron variants became the dominant strain in Toronto |
| **Post-Omicron period** | Outcomes occurring after December 31 2021, after Omicron variants became the dominant strain in Toronto |

**References**

1. Benchimol EI, Smeeth L, Guttmann A, Harron K, Moher D, Petersen I, Sørensen HT, von Elm E, Langan SM, RECORD Working Committee. The REporting of studies Conducted using Observational Routinely-collected health Data (RECORD) statement. *PLoS medicine*. 2015 Oct 6;12(10):e1001885.
2. Richard L, Nisenbaum R, Colwill K, Mishra S, Dayam RM, Liu M, et al. Enhancing detection of SARS-CoV-2 re-infections using longitudinal sero-monitoring: demonstration of a methodology in a cohort of people experiencing homelessness in Toronto, Canada. *BMC Infectious Diseases*. 2024 Feb 2; 24(1):125
